# WHERE ARE THE ORTHOPEDIC ONCOLOGY CENTERS IN THE BRAZILIAN UNIFIED HEALTH SYSTEM (SUS)?

**DOI:** 10.1590/1413-785220253306e293805

**Published:** 2025-11-10

**Authors:** Geraldo Mota Gonçalves, Danilo Arruda de Souza, Edgard Eduard Engel

**Affiliations:** 1 Universidade de Sao Paulo (USP), Faculdade de Medicina de Ribeirao Preto, Ribeirao Preto, Sao Paulo, SP, Brazil.

**Keywords:** Health Services Accessibility, Cancer Care Facilities, Bone Neoplasms, Orthopedics, Acesso aos Serviços de Saúde, Institutos de Câncer, Neoplasias Ósseas, Ortopedia

## Abstract

**Objective:**

To identify and analyze the geographical distribution, surgical volume, and population adequacy of Orthopedic Oncology Centers (OOCs) within the Brazilian Unified Health System (SUS).

**Methods:**

We evaluated 11,139 procedures recorded in Hospitalization Authorizations (AIHs) between 2008 and 2019, including "hemipelvectomy in oncology" and "resection of bone tumors with replacement or reconstruction." Hospitals performing both procedures and at least three hemipelvectomies during this period were classified as OOCs.

**Results:**

A total of 58 OOCs were identified in 18 states, accounting for 79.5% of all procedures. Most patients (93.7%) were treated in their home state. High-Volume Centers (HVCs) performed 95% of the surgeries, while Low-Volume Centers (LVCs) were responsible for only 5%. The Northeast region concentrated 39.1% of procedures, while the South had the highest number of OOCs per population.

**Conclusion:**

The geographical distribution of OOCs in Brazil is relatively adequate; however, procedures are highly concentrated in a few high-volume centers. This centralization may be associated with better clinical outcomes, reinforcing the need for policies that encourage specialized treatment in reference units. ***Level of Evidence III; Cross-Sectional Observational Study***.

## RESUMO

**Objetivo:**

Identificar e analisar a distribuição geográfica, o volume cirúrgico e a adequação populacional dos Centros de Oncologia Ortopédica (COOs) no Sistema Único de Saúde (SUS).

**Métodos:**

Foram avaliados 11.139 procedimentos registrados em Autorizações de Internação Hospitalar (AIHs) entre 2008 e 2019, correspondentes a "hemipelvectomia em oncologia" e "ressecção de tumor ósseo com substituição ou reconstrução". Consideraram-se COOs os hospitais que realizaram ambos os procedimentos e pelo menos três hemipelvectomias no período.

**Resultados:**

Foram identificados 58 COOs em 18 estados, responsáveis por 79,5% dos procedimentos. A maioria dos pacientes (93,7%) foi tratada em seu estado de origem. Centros de Alto Volume (CAV) realizaram 95% das cirurgias, enquanto Centros de Baixo Volume (CBV) responderam por apenas 5%. O Nordeste concentrou 39,1% dos procedimentos, e o Sul apresentou o maior número de COOs por habitante.

**Conclusão:**

Existe distribuição geográfica relativamente adequada dos COOs no Brasil, mas com grande concentração de procedimentos em poucos centros de alto volume. Essa centralização pode estar associada a melhores desfechos clínicos, reforçando a necessidade de políticas que incentivem a consolidação do tratamento em unidades especializadas. ***Nível de Evidência III; Estudo Observacional Transversal***.

## INTRODUCTION

In Brazil's Unified Health System (SUS), the healthcare network is stratified and divided into Health Regions, whose purpose is to ensure medical care at all levels of complexity for the entire population.^1^ The rarer the disease, the fewer the number of referral centers available within the network, and the greater the distance patients must travel to receive specialized care.

Musculoskeletal malignancies are rare, accounting for less than 1% of neoplasms in adults and 15% in children.^2^ Delays in diagnosis profoundly impact patient prognosis, often resulting in mutilating surgeries or low survival rates. In developed countries, the average time between the first symptom and diagnosis is six weeks; for pelvic bone tumors, this interval increases to 16 weeks. In Brazil, it is estimated that the time is twice as long.^3^ In a study conducted in India, difficulty accessing orthopedic oncology centers—defined as delayed diagnosis or treatment—was observed in 72.2% of cases.^4^

The literature does not provide a clear definition of an Orthopedic Oncology Center (OOC), but it is well established that such centers should have a multidisciplinary team with specialized physicians, infrastructure with advanced diagnostic and treatment resources, and preferably a high patient flow that ensures extensive experience in the field.^5,6^

In Brazil, both physicians and patients face significant challenges in identifying such centers. The National Cancer Institute (INCA) lists 317 hospital units accredited for cancer treatment, in which the participation of an orthopedic specialist on the medical team is required.^1,7,8^ However, there is no requirement that this orthopedic surgeon have specific training in orthopedic oncology.

These institutions are divided into two main models: UNACON (High-Complexity Oncology Units), which must provide care for the most prevalent cancers, and CACON (High-Complexity Oncology Centers), which must treat all cancer types and provide in-house radiotherapy services.^1,8^ According to recent studies, the number of such hospitals is insufficient, and their geographic distribution is inadequate.^9^

Therefore, the aim of this study was to identify Orthopedic Oncology Centers within the Brazilian Unified Health System and to assess their geographic distribution, surgical volume, and population adequacy.

## METHODOLOGY

This study was approved by the local ethics and research committee under certificate number 125276/2023. Data from Hospital Admission Authorizations (AIHs), contained in the Hospital Information System of the Unified Health System (SIHSUS), were analyzed between January 2008 and December 2019. These data originate from the files of the Brazilian Ministry of Health, managed by the Department of Informatics of the Unified Health System (DATASUS), and are used for accountability between hospitals and the Ministry of Health.

The tools Dbsaúde® (Numb3rs Analytics®, 2017, Barueri, Brazil) and Tableau Software® (Salesforce Brasil®, 2019, São Paulo, Brazil) were used to process the data by the Executive Agreement Group of the Hospital das Clínicas, Ribeirão Preto Medical School, University of São Paulo (HC FMRP-USP). From the AIHs, the following data were extracted: (1) CNES – National Registry of Health Establishments, corresponding to the hospital name recorded in the DATASUS database; (2) municipality and (3) state of the establishments that issued the AIH; (4) municipality and (5) state of residence of the patient who underwent the procedure; (6) the date, including month and year, of the procedure; and (7) the AIH number.

The only procedures available in the AIHs that are exclusively related to the orthopedic oncology specialist and performed in high-complexity hospitals are "hemipelvectomy in oncology" and "resection of bone tumor with substitution (endoprosthesis) or with reconstruction and fixation in oncology," coded in the Sigtap (Management System of the Table of Procedures, Medications, and OPM of the SUS).^10^ This field was used as a filter to identify Orthopedic Oncology Centers (OOCs) in Brazil.

The 11,139 procedures identified (745 hemipelvectomies and 10,394 oncologic resections) were carried out in 205 facilities across 27 states in 121 municipalities. These procedures were performed on patients from 2,446 municipalities across the 27 states. Since many of these facilities performed the procedures randomly or sporadically, a subjective eligibility criterion was created to define OOCs. Hospitals that, between 2008 and 2019, performed at least three hemipelvectomies and one bone resection with substitution or reconstruction in oncology were selected.

After applying this selection criterion, a total of 8,861 procedures performed in 58 hospitals were obtained, corresponding to 79.5% and 28.2% of the initial dataset, respectively. These hospitals, classified as OOCs, were cross-referenced with their respective SUS accreditations,^7^ regarding certification as CACON (High-Complexity Oncology Centers) or UNACON (High-Complexity Oncology Units, with or without pediatric oncology care), and with national, macroregional, and state population estimates from the Brazilian Institute of Geography and Statistics (IBGE) for 2019.^11^

## RESULTS

### Identification of Orthopedic Oncology Centers

The survey enabled the development of a catalog containing the names of the establishments according to CNES, their accreditation for oncology care within the SUS,^7^ location, and provision of pediatric oncology and radiotherapy services. These parameters allowed for both qualitative and quantitative assessments of the centers, analyzed collectively and individually. Of the 58 identified OOCs, 26 were CACONs, 18 of which provided pediatric care. The remaining 32 corresponded to UNACONs, of which 15 offered pediatric care, including two centers dedicated exclusively to pediatric care: Boldrini Campinas and Hospital GRAACC Instituto de Oncologia Pediátrica (IOP).

### Geographic Distribution

The OOCs are distributed across 18 states and 37 municipalities. These centers served patients from 2,001 cities covering all 27 Brazilian states. The state of São Paulo accounted for 29.3% of these centers, followed by Minas Gerais and Paraná, with 10.3% each, and Rio Grande do Sul, Santa Catarina, and Pernambuco, with 6.9% each (Table 1). On the other hand, 9 states did not have OOCs according to the criteria applied in this study, which resulted in greater patient migration in search of specialized care in other states or regions. Five of these states are located in the North region, two in the Northeast, and two in the Center-West.

**Table 1:** Distribution of Orthopedic Oncology Procedures by State and Population Density.

State	Population	%	Centers	%	Procedures	%	OOC/M. inhab.	Procedures/M. inhab.
SP	45,919,049	21.9	17	29.3	2207	24.9	0.37	48
MG	21,168,791	10.1	6	10.3	812	9.2	0.28	38
RJ	17,264,943	8.2	1	1.7	169	1.9	0.06	10
BA	14,873,064	7.1	2	3.4	1055	11.9	0.13	71
PR	11,433,957	5.4	6	10.3	811	9.2	0.52	71
RS	11,377,239	5.4	4	6.9	479	5.4	0.35	42
PE	9,557,071	4.5	4	6.9	1039	11.7	0.42	109
CE	9,132,078	4.3	3	5.2	779	8.8	0.33	85
PA	8,602,865	4.1	2	3.4	138	1.6	0.23	16
SC	7,164,788	3.4	4	6.9	122	1.4	0.56	17
GO	7,018,354	3.3	1	1.7	333	3.8	0.14	47
AM	4,144,597	2.0	1	1.7	141	1.6	0.24	34
ES	4,018,650	1.9	1	1.7	57	0.6	0.25	14
PB	4,018,127	1.9	1	1.7	246	2.8	0.25	61
RN	3,506,853	1.7	1	1.7	159	1.8	0.29	45
PI	3,273,227	1.6	1	1.7	172	1.9	0.31	53
DF	3,015,268	1.4	2	3.4	128	1.4	0.66	42
SE	2,298,696	1.1	1	1.7	14	0.2	0.44	6
BRAZIL	210.147.125	100	58	100.0	8861	100.0	0.28	42
SD							0.15	27

OOC: Orthopedic Oncology Center according to this study's criteria. M. inhab.: million inhabitants. SD: standard deviation. States without OOC: Acre; Alagoas; Amapá; Maranhão; Mato Grosso; Mato Grosso do Sul; Rondônia; Roraima; Tocantins.

All five Brazilian macro-regions are covered by OOCs, with the following distribution: 43.1% in the Southeast, 24.1% in the South, 22.4% in the Northeast, 6.9% in the Center-West, and 3.4% in the North. (Table 2)

**Table 2:** Distribution of Orthopedic Oncology Procedures by Macro-Regions and Population Density.

Region	Population	%	Centers	%	Procedures	%	OOC/M. de inhab.	Procedures/M. de inhab.
Southeast	88,371,433	42.1	25	43.1	3,245	36.6	0.28	37
Northeast	57,071,654	27.2	13	22.4	3,464	39.1	0.23	61
South	29,975,984	14.3	14	24.1	1,412	15.9	0.47	47
North	18,430,980	8.8	2	3.4	279	3.1	0.11	15
Center-West	16,297,074	7.8	4	6.9	461	5.2	0.25	28
Brazil	210,147,125	100	58		8,861		0.28	42
SD							0.12	16

OOC: Orthopedic Oncology Center according to this study's criteria. M. inhab.: million inhabitants. SD: standard deviation.

### Geographic Reach of the Centers

**Figure 1:**
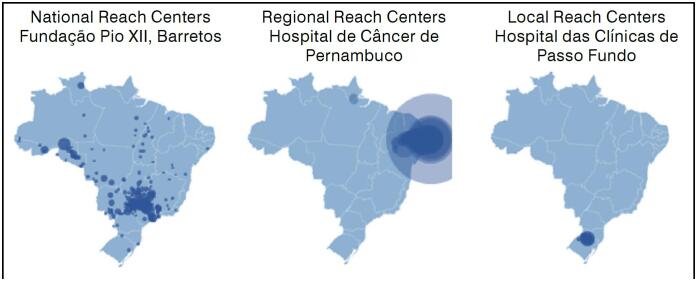
Maps of the reach profiles of orthopedic oncology centers in Brazil.

The survey of the municipalities of origin of the patients treated at each center allowed the creation of maps showing the catchment areas of each OOC. Some centers receive patients from up to 16 different states. Conversely, the vast majority serve patients from their own state. Three distinct profiles were identified: National reach centers, which serve a large region beyond state boundaries, such as Fundação Pio XII in the city of Barretos; Regional reach centers, which cover a macro-region, with few long-distance cases and a high volume of procedures; Local reach centers, which serve patients from a small region, with shorter distances and reduced patient flow. (Figure 1)

Bubble location: Origin of patients treated by the OOC. Bubble size: Number of patients from each locality treated

### Surgical Volume Analysis of Orthopedic Oncology Centers

When dividing the OOCs into quartiles, it was observed that the 15 hospitals in the first quartile were responsible for 63.8% of the procedures included in the study, and the first two quartiles together accounted for 86.9% of the procedures (or 75.6% of the initial total). The lowest-volume quartile, consisting of 14 hospitals, performed only 2.6% of the procedures analyzed.

A significant discrepancy in the number of surgeries performed was identified, allowing the classification of OOCs into High-Volume Centers (HVCs), which include the first quartile, and Low-Volume Centers (LVCs), corresponding to the quartile with the lowest number of procedures.

There was a trend for HVCs to be accredited as CACONs and LVCs as UNACONs. However, this was not an absolute rule, as the second highest volume identified was from the Hospital de Câncer de Pernambuco, a UNACON. Conversely, among the 51 CACONs registered in the SUS, 25 were not classified as OOCs in this study.

Over the 12 years analyzed, 95% of the procedures were performed in 42 OOCs, while the remaining 16 centers accounted for only 5% of the total. The macro-regions with the highest concentration of procedures were the Northeast (39.1%), followed by the Southeast (36.6%), South (15.9%), Midwest (5.2%), and North (3.1%). (Table 2)

### Adequacy of Orthopedic Oncology Centers to the Population

#### Macro-regions

**Figure 2:**
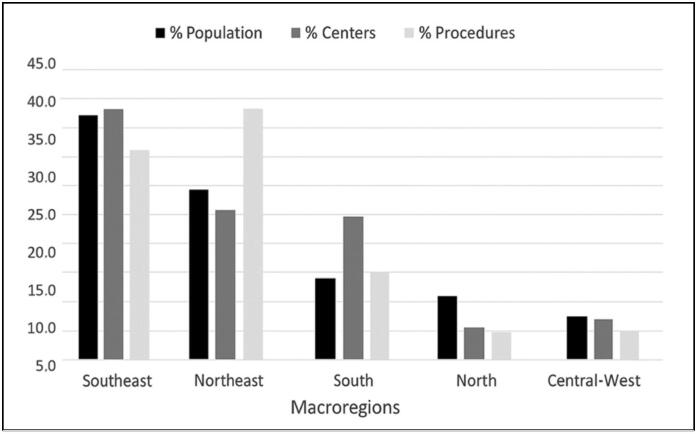
Relationship between the proportions of population, number of OOCs, and procedures in the macro-regions.

Table 2 presents a summary of the correlation between population density, the number of OOCs, and the procedures performed. In the Southeast and Midwest regions, the proportions of these variables are similar to the national average and considered balanced. In the Northeast, a disproportionately high number of procedures (39.1%) was observed relative to its population (27.2%) and number of centers (22.4%). In contrast, in the South, a high number of OOCs (24.1%) was identified compared to its population (14.3%) and the number of surgeries performed (15.9%). Finally, in the North, both the number of OOCs (3.4%) and procedures (3.1%) were relatively low compared to the population (8.8%). (Figure 2)

These findings are confirmed when using the coefficients of centers and procedures per million inhabitants. The Northeast showed a high coefficient of procedures per inhabitant, while the South presented elevated coefficients for both centers and procedures. The North had below-average coefficients, and the Southeast and Midwest demonstrated values close to the Brazilian average.

### Federative Units

In the state-level analysis (Table 1), the low number of centers (1.7%) and procedures (1.9%) in the state of Rio de Janeiro stands out when compared to its population (8.2%). On the other hand, Pernambuco, Bahia, and Ceará presented a high surgical volume relative to their local demographic proportion. Pernambuco accounts for 4.5% of the population, yet concentrates 6.9% of the COOs and 11.7% of the procedures. Bahia, with 7.1% of the population and 3.4% of the COOs, performed 11.9% of the surgeries analyzed. Ceará, with 4.3% of the population, accounted for 5.2% of the COOs and 8.8% of the surgeries. São Paulo, Paraná, and Paraíba are also states in which the proportion of procedures is greater than the share of the population.

**Figure 3:**
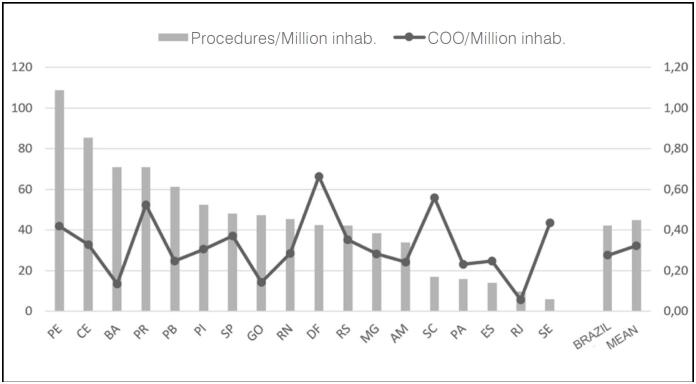
Relationship between the coefficients of Centers and Procedures per million inhabitants in the Federative Units.

Regarding the coefficient of COO concentration (Figure 3), the Federal District and the states of Santa Catarina and Paraná presented the highest values. In Paraná, this coefficient was accompanied by a high coefficient of procedures. At the other extreme, Rio de Janeiro, Bahia, and Goiás had fewer COOs relative to their populations than the Brazilian average.

Pernambuco and Ceará showed the highest coefficients of procedures, remaining above one standard deviation from the national average (42 procedures per million inhabitants). Conversely, Santa Catarina, Pará, Espírito Santo, Rio de Janeiro, and Sergipe were below one standard deviation (Table 1). The chart in Figure 3 shows that there is no correlation between the coefficient of centers and procedures, and that there is wide variation in relation to the national average.

### Analysis of Migrations

The correlation between the patient's state of origin and the state where treatment was performed allowed us to evaluate the migrations that occurred and the capacity of each state to treat its own patients. In 9 states, patient migration is mandatory, or procedures are carried out in hospitals not classified as COOs.

Patients from the Southeast, South, and Northeast remain within their macroregions, with resolution rates close to 100%. In the Midwest and North regions, migratory flows correspond to 20.4% and 30.1% of patients, respectively. However, these rates represent only a small portion of patients at the national level. Thus, the percentage of patients who migrated to another macroregion was only 3.3%.

States without COOs, which correspond to 10.6% of the population, accounted for 39.4% of interstate migrations. In contrast, among states with COOs, only Sergipe did not show a resolution rate higher than 80% within its own state. Overall, the rate of treatment resolution within the patient's home state was above 90%.

Qualitative analysis revealed preferential migration flows. In states without COOs, São Paulo was the main destination, receiving most patients from Acre, Roraima, Rondônia, Tocantins, Mato Grosso do Sul, and Mato Grosso. Patients from Amapá migrated preferentially to Pará, those from Maranhão to Piauí, and those from Alagoas to Pernambuco.

## DISCUSSION

The national scientific literature lacks information on patient flow within the Brazilian Unified Health System (SUS). The development of healthcare strategies and the rational distribution of resources across the country are only possible with a deep understanding of the installed infrastructure and the availability of human resources. In the case of orthopedic oncology, as in other high-complexity areas, infrastructure requires high investment in technology, and human resources demand prolonged training.

The quality of data analysis from a database is closely linked to the quality of the database itself. In the case of DataSUS, the following factors may have impacted the results:

- Hospital Admission Authorizations (AIHs) may not have been completed in some hospitals because they exceeded the SUS budget cap, were denied reimbursement, or received funding from another payer other than SUS.
- Codes may have been incorrectly recorded, either by the physician or by administration.
- The residence address may have been altered by the patient, with some frequency, to allow treatment in the hospital of choice.

Among these, two factors appear most significant: 1) the migration index may be higher than that presented in this study, and 2) the volume of care in hospitals in Rio de Janeiro may be underestimated, possibly due to the financing model.

Since there is no formal definition of an Orthopedic Oncology Center (COO), an arbitrary criterion was used. Hemipelvectomies are uncommon procedures but have high specificity for orthopedic oncology.^7^ The procedure "resection of bone tumors and reconstruction" is also specific, though more common. With the exception of small resections of the iliac wing, both procedures require a professional trained in orthopedic oncology. Although three hemipelvectomies and one bone reconstruction in 12 years represent a low surgical volume, these numbers do not reflect the total number of procedures performed in orthopedic oncology and should therefore be considered as a sample of procedures performed in each COO.

With this less restrictive criterion, a greater number of hospitals were included, accounting for nearly 90% of the procedures selected during the study period, thereby allowing a broader and more detailed analysis.

Despite the limitations introduced by these factors, the analysis of 11,139 surgical procedures typical of orthopedic oncology, over a 12-year period, certainly provides consistent results and reflects the reality of public care for musculoskeletal cancer in Brazil.

Several authors confirm that specialized high-volume centers achieve the best outcomes in the treatment of sarcomas.^5,6,12-15^ Well-structured orthopedic oncology groups, which sometimes act as regional references and operate in more than one hospital, may have been excluded, while other less specialized hospitals may have been included. On the other hand, the definition of a COO is not based solely on surgical volume. The care of malignant musculoskeletal tumors is multidisciplinary and requires several additional components that were not addressed in this study.^15^

Although oncology care hospitals accredited by SUS are clearly identified as CACON (51) and UNACON (263), the study demonstrated that not all of them provide care for musculoskeletal tumors. Even the regulations requiring the inclusion of orthopedic surgeons in High-Complexity Oncology Centers and Units do not mandate that these professionals be trained in orthopedic oncology. This is compounded by the fact that orthopedic oncology is not formally recognized as a specialty or subspecialty by SUS. Conversely, if all these centers and units provided orthopedic oncology care, services would be dispersed across 314 centers, when it is already known that 41 centers are capable of handling 75.6% of the national demand, and high surgical volume is associated with better outcomes. This suggests that a regulatory change would be the most appropriate solution.

The vast majority of surgeries were performed either within the patient's state (93.7%) or within the same macroregion (96.7%), indicating low migration rates. Even considering the potential bias of falsified residence declarations, these values can be considered very high and reflect the quality of the various centers across Brazil, whose geographic distribution correlates reasonably well with the population density of each macroregion.

In summary, the methodology used identified hospitals that provide specialized orthopedic oncology care within SUS. A total of 58 Orthopedic Oncology Centers (COOs) were identified, which accounted for 8,861 of the 11,139 (79.5%) procedures included in the study.

Regarding surgical volume, 95% of the selected procedures were performed in 42 of the 58 COOs studied, while 2,721 procedures (24.4% of the total) were performed in low-volume hospitals on a sporadic basis.

The orthopedic oncologist is a rare professional who treats a rare disease. On the one hand, there should not be an excessive number of bone cancer treatment centers; on the other hand, Brazil's size requires a geographic distribution that ensures patient access. The study also suggests that mechanisms should be created to concentrate musculoskeletal tumor cases in institutions led by trained orthopedic oncologists, avoiding the sporadic performance of highly complex procedures by less experienced professionals.

This study provides important data and may serve as a valuable tool for healthcare managers, as well as a basis for discussing adjustments in the public healthcare system for rare diseases, such as orthopedic oncology.

In conclusion, according to the criteria used, 58 Orthopedic Oncology Centers (COOs) were identified, which accounted for 79.5% of the 11,139 procedures analyzed in the study.

The geographic distribution of COOs correlates reasonably well with the distribution of the Brazilian population and ensures a low interstate migration rate (6.3%). On the other hand, the variation across states in the number of procedures per million inhabitants is considerable (ranging from 6 to 109, with an average of 42), and the main cause of this variation is the quality of information recorded in the AIHs.

There is a tendency toward concentration of procedures in high-volume COOs, since 95% of procedures were carried out in 42 of the 58 COOs. This trend aligns with the literature, which associates high-volume centers with better clinical outcomes. However, Brazilian legislation lacks mechanisms to consolidate this trend, which would promote specialized treatment for musculoskeletal tumors, as well as for other rare diseases.
